# Mesenchymal Stem Cells and Induced Pluripotent Stem Cells as Therapies for Multiple Sclerosis

**DOI:** 10.3390/ijms16059283

**Published:** 2015-04-24

**Authors:** Juan Xiao, Rongbing Yang, Sangita Biswas, Xin Qin, Min Zhang, Wenbin Deng

**Affiliations:** 1 Medical College, Hubei University of Arts and Science, Xiangyang 441053, China; E-Mails: ju_126@126.com (J.X.); qinxin@fmmu.edu.cn (X.Q.); minzhang@ucdavis.edu (M.Z.); 2 Department of Biological Treatment, Handan Central Hospital, Handan 056001, China; E-Mail: zmhbuas@outlook.com; 3 Department of Biochemistry and Molecular Medicine, School of Medicine, University of California, Davis, CA 95817, USA; E-Mail: sangita.biswas@ucdmc.ucdavis.edu

**Keywords:** mesenchymal stem cells, induced pluripotent stem cells, neural stem cell, multiple sclerosis

## Abstract

Multiple sclerosis (MS) is a chronic, autoimmune, inflammatory demyelinating disorder of the central nervous system that leads to permanent neurological deficits. Current MS treatment regimens are insufficient to treat the irreversible neurological disabilities. Tremendous progress in the experimental and clinical applications of cell-based therapies has recognized stem cells as potential candidates for regenerative therapy for many neurodegenerative disorders including MS. Mesenchymal stem cells (MSC) and induced pluripotent stem cell (iPSCs) derived precursor cells can modulate the autoimmune response in the central nervous system (CNS) and promote endogenous remyelination and repair process in animal models. This review highlights studies involving the immunomodulatory and regenerative effects of mesenchymal stem cells and iPSCs derived cells in animal models, and their translation into immunomodulatory and neuroregenerative treatment strategies for MS.

## 1. Introduction

Multiple sclerosis (MS) is a chronic autoimmune, inflammatory, demyelinating, neurodegenerative disease of the central nervous system (CNS) of unknown etiology. The major clinical subtypes of MS are relapsing-remitting (RR) and secondary progressive (SP) MS. Clinically, RRMS represents the initial inflammatory phase characterized by partially reversible symptomatic attacks with temporary loss of motor functions, followed by periods of remission [1]. Approximately 50%–60% of these patients progress to SPMS in which the disease gradually evolves from intermittent attacks to slow, steady progressive worsening condition ultimately resulting in permanent disabilities resulting from massive axonal loss (seen in autopsy tissue sections). Primary progressive (PP) MS, the most severe subtype of MS, affects approximately 10% of all cases and manifests as progressive neurological decline in function from disease onset without remission [2]. The etiology of MS is believed to involve interplay between environmental factors such as vitamin D deficiency, UV light exposure, Epstein-Barr virus (EBV) infection [3], and individual genetic susceptibility [4].

## 2. Current Concepts in Multiple Sclerosis (MS) Pathology

Pathological hallmarks of MS and experimental autoimmune encephalomyelitis (EAE) include CNS inflammation, gliosis, demyelination, and axonal loss. CNS pathology in patients is characterized by multifocal lesions, the MS plaques. In the acute phase, activated mononuclear cells, including lymphocytes, microglia, and macrophages destroy myelin and to a variable degree, oligodendrocytes. With time, gliosis develops, and plaques reach a burned-out stage consisting of demyelinated axons traversing glial scar tissue. The endogenous oligodendrocyte precursor cells (OPCs) proliferate robustly in response to demyelination, but fail to differentiate terminally, resulting in failure of remyelination. This is associated with axonal transection leading to permanent neurological deficits. If the inflammatory process is arrested at an early phase, plaques are partially remyelinated. Autopsy samples revealed that SPMS is associated with massive axon loss in the CNS and unmyelinated plaques [5,6].

Multiple cell types, including type 17 T helper cells (TH17), forkhead box P3 (FOXP3) regulatory T cells, B cells and macrophages are involved in MS disease pathogenesis [7]. It has been hypothesized that the first event in the pathogenesis of MS could be a viral infection in infancy. Activated T-lymphocytes generated during such an infection cross the blood-brain barrier and become sensitized to myelin antigens. Alternatively, lymphocytes are sensitized to viral proteins that share homology to myelin proteins. It is speculated that, after remaining latent for years, these autoreactive T cells re-enter the CNS through the impaired blood-brain barrier (BBB) and initiate an immune reaction against myelin. B-lymphocytes entering acute plaques become sensitized and produce antibodies to myelin antigens. Myelin, oligodendroglial cells, and axons are damaged by inflammatory cytokines, glutamate, nitric oxide (NO), and other toxic substances produced by microglia/macrophages. While the agents of CNS damage are the same, the immune reactions that initiate and propagate the process may vary among different forms of MS.

Currently, there is no cure for MS. During the past decades, therapies for MS are either immunomodulatory or immunosuppressive [8]. Immunomodulatory and anti-inflammatory agents are effective in the relapsing–remitting stage by reducing the frequency of relapses, and decreasing the formation of inflammatory lesions [9], but they do not influence the course of progressive MS and therefore are not sufficient enough to cure chronic neurological disability. The permanent neuronal loss that starts early and characterizes the progressive stage of MS remains untreatable. Therapeutic options such as Mitoxantrone for PPMS patients are limited to symptomatic treatments and the long-term prognosis is generally poor [10]. Future therapeutic strategies are aimed to achieve neuroprotection, remyelination and regeneration of new oligodendrocytes and neurons [11].

## 3. Stem Cells as a “Biological Solution to a Biological Problem”

Stem cells are unspecialized cells in the body that retain the ability to generate cells of undifferentiated state identical to themselves, or of differentiating into other types of body cells with specialized functions [12]. There are various types of stem cells such as embryonic stem cells (ESCs), hematopoietic stem cells (HSCs), neural stem cells (NSCs), mesenchymal stem cells (MSCs) and induced pluripotent stem cells (iPSCs) [13]. Nowadays, with the great progression of biology and biotechnology, we are exploring “biological solutions to biological problems”. Stem cell therapy in axonal demyelination and neurological disability has had promising results in animal models as well as human patient clinical treatment [14]. Stem cell therapies may serve as potential therapy for neurodegenerative disease. Here, we review the recently published studies regarding preclinical and clinical use of MSCs and iPSCs in the treatment of MS, and their therapeutic mechanisms. Among the various types of stem cells, the efficacy and safety of MSCs have long been well established and characterized, but the recent advancements of iPSCs make them a promising candidate for autologous therapy. Both MSCs and iPSCs can be obtained from patients relatively easily and can be expanded for use.

## 4. Mesenchymal Stem Cells (MSCs)

MSCs were first isolated from bone marrow, characterized, and described by Friedenstein *et al.* in 1968. These spindle-shaped, fibroblastic cells constitute about 0.01% to 0.001% of the entire nucleated cells of bone marrow. Caplan *et al.* introduced the term “mesenchymal stem cells” as they could be induced *in vitro* and *in vivo* to differentiate into other lines of mesodermal cells, including adipocytes, chondrocytes, connective stromal cells, and osteocytes-cells [15]. MSCs can be isolated from various sources such as bone marrow, amniotic fluid, dental pulp, adipose tissue [16], umbilical cord [17], synovial membranes, and peripheral blood, among which the main and the most frequently studied source is the bone marrow. MSCs are characterized by (i) the positive expression of CD105, CD90 and CD73 and negative expression for haematopoietic cell surface markers CD34, CD45, CD11a, CD19 or CD79a, CD14 or CD11b, and human leukocyte antigen-DR (HLA-DR); (ii) under a specific stimulus, MSCs differentiate into osteocytes, adipocytes and chondrocytes *in vitro*. These criteria are currently used to purify MSC and to enable their expansion *in vitro*, without losing their differentiation capacity [18]. It has been recently reported that MSCs are able to differentiate into non-mesenchymal cell lineages, such as skeletal myocytes, neurons and cells of the visceral mesoderm, both *in vitro* and *in vivo* [17,19].

## 5. Efficacy of MSCs in Mouse Experimental Autoimmune Encephalomyelitis (EAE) Mouse: Current Evidence

In the EAE mouse model of multiple sclerosis, MSCs systematically injected at disease onset ameliorates myelin oligodendrocyte glycoprotein (MOG)-induced EAE, and decreases the infiltration of T-cells, B-cells and macrophages into the brain and spinal cord. MSCs can cause induction of T-cell anergy, since T cells extracted from the lymph nodes of MSC-treated mice are unable to proliferate after *in vitro* re-challenge with MOG peptide [20]. Systematic injection of MSCs can inhibit the *in vivo* production of pathogenic proteolipid protein (PLP)-specific antibodies and to suppress the encephalitogenic potential of PLP-specific T cells in passive-transfer experiments. The MSCs migrated to the spleen, as well as, to the inflamed CNS, where they exercised a neuroprotective effect on the axons [21]. In these studies, the therapeutic effect of MSCs depended on the release of anti-apoptotic, anti-inflammatory and trophic molecules, and, possibly, on the recruitment of local progenitors and their subsequent induction to differentiate into neural cells. As a trophic effect, the MSCs appeared to favor oligodendrogenesis by neural precursor cells [22]. However, recent reports also indicate that MSCs possess duality in immunomodulation [23,24] and even exacerbate the symptoms. In a pathogenic CD8^+^ T cells mediated MOG model of experimental autoimmune encephalomyelitis (EAE), a commonly used murine model of MS, MSCs deteriorated the disease and increased the CD8^+^ T cell presence in the brains of diseased mice [25].

**Keypoints**: Bone marrow (BM)-derived MSCs attenuate PLP and MOG induced EAE by suppressing PLP and MOG specific autoreactive T cells.

## 6. Effect of the Inflammatory Environment of EAE on Endogenous MSCs

It appears that the inflammatory environment imposes certain impact on BM-MSCs despite that BM-MSCs residing in the bone marrow are not directly implicated in the disease process. BM-MSCs isolated from EAE mice exhibited distinct morphology, elevated ratio of proliferation and apoptosis, differences in the adipogenesis and the osteogenesis induction, distinct expression profile of stromal markers [26] and different expression patterns on six histone-modifying genes compared to MSCs from control mice [27]. However, another report indicated that the inflammatory process did not exert any deleterious effect on the functional/biological properties of the BM-MSCs isolated from mice with EAE [28]. Intravenous administration of congenic BM-MSCs derived from EAE mice suppressed EAE development in transplanted mice, along with remarkable reduction of CNS inflammation and demyelination and, protection of the axons. There were no significant differences in these beneficial effects between EAE-BM-MSCs and MSCs obtained from wild-type syngeneic donors. These data showed conflicting findings regarding the therapeutic effectiveness of autologous BM-MSCs.

In a recent study, adipose stromal/stem cell (ASCs) from mice with EAE and their syngeneic wild-type mice were cultured and expanded *in vitro* under standard cell culture condition. Although EAE-ASCs displayed a normal phenotype with typical MSCs surface antigen expression, they showed no therapeutic improvement on the disease progression *in vivo*. However, infusion of wild-type ASCs significantly ameliorated the disease course. Experimental results showed that EAE-ASCs mice acquired increased expression of monocyte chemoattractant protein-1 (MCP-1), a pro-inflammatory cytokine [29]. MCP-1 can modulate the blood-brain barrier disruption and mononuclear leukocyte migration into the CNS [30]. It appears that the EAE-ASCs may have been irreversibly altered in the setting of chronic neuroinflammation and lost their therapeutic efficacy for the treatment of EAE mice. Mechanisms underlying the therapeutic discrepancy between MSCs obtained from different sources need further intensive research in the future to provide valuable information for autologous MSCs transplantation for the treatment of MS patients.

However, EAE does not reflect all aspects of MS pathology, and murine derived MSCs do not share exactly the same properties with those of human origin. Additionally, MSCs in human studies were derived from MS patients where the inflammatory process was not comparable with those induced in acute EAE. Previous studies suggested that MSCs from patients with MS display a normal phenotype and are fully functional in terms of proliferation, *in vitro* differentiation and immunosuppressive capacity [31].

## 7. Efficacy of Genetically Engineered Human MSCs in Mouse EAE Models

Human MSCs genetically engineered to over-express the anti-inflammatory cytokines promote curative effect in EAE models. IFN-β has a potent anti-inflammatory effect and has been used to treat RRMS for nearly two decades. Human BM-MSCs engineered to secret IFN-β (MSCs-IFN-β) via adenoviral transduction outperformed MSCs alone in decreasing inflammatory infiltration and demyelination in the lumbar spinal cord and inhibition of mice EAE onset [32]. MSCs-IFN-β exhibited augmented immunomodulatory effects and reduced further injury of BBB permeability in EAE mice via migrating into inflamed CNS [32]. Other cytokines also were exploited to strengthen the therapeutic effectiveness of MSCs. For example, human adipose-derived MSCs (Adi-MSCs) were engineered to over-express IL-4 attenuated symptoms in mice with EAE when administered early at the time of T-cell priming. The therapeutic efficacy was assumed to associate with a reduction in peripheral autoreactive T-cell responses and a shift from a pro- to an anti-inflammatory cytokine response [33]. Another study showed that Adi-IL-10-MSCs could prevent or delay the development of EAE if transplanted via intraperitoneal route early in the disease course to mice. An important mechanism involved might be inhibition of the maturation, cytokine expression profile and antigen presenting capacity of bone marrow-derived myeloid dendritic cells [34]. More cytokines and molecules such as interleukins, chemokines and their cognate receptors could be employed via transgenic technique to cooperate with the effects of MSCs either by their existence or absence.

## 8. Mechanisms of Action of MSCs

Much research has focused on exploiting the pleiotropic properties of MSCs as a basis for cell therapy for a variety of neurodegenerative disorders including MS [19]. The immunomodulatory, immunosuppressive, neurotrophic, and repair-promoting properties of MSCs [35] make them an attractive candidate for MS. The effects of MSCs on immunomodulation and remyelination are largely mediated by paracrine signals [22], and several secreted soluble molecules [20], TGF-β1 [36], IFN-γ [37], indoleamine 2,3-dioxygenase (IDO) and prostaglandin E2, have been identified as important contributors to these beneficial effects.

## 9. Immunosuppressive Effects of MSCs

**Figure 1:**
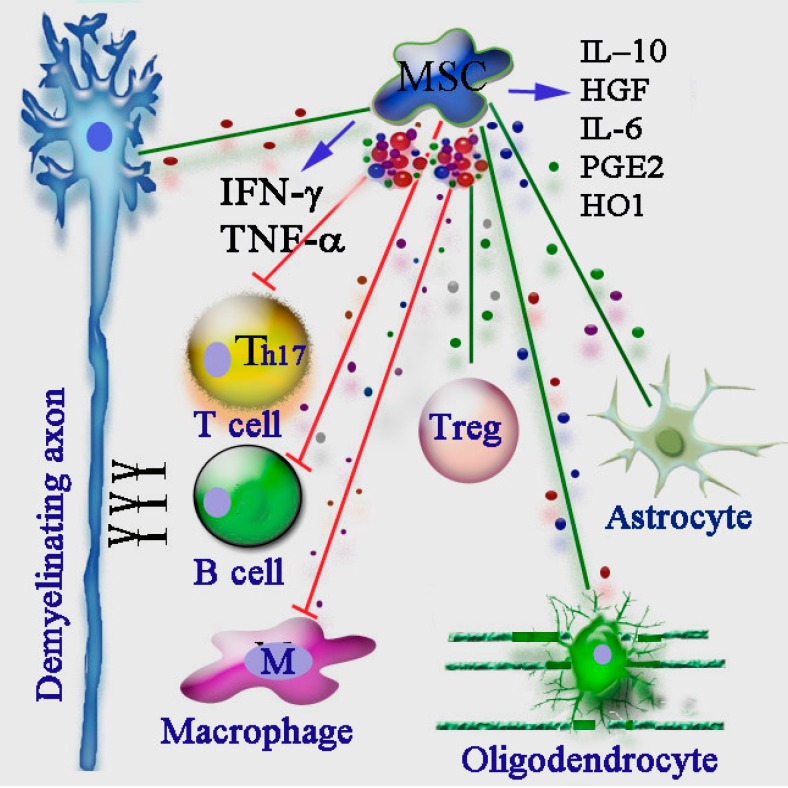
The complex immunomodulatory functions of mesenchymal stem cells (MSCs) in the treatment of multiple sclerosis (MS). The different immune regulatory mechanisms, including pathogenic B cells, Th17 cells, macrophages, and beneficial regulatory T cells (Treg) are involved in shaping the MS pathology. Injection of MSCs improves experimental autoimmune encephalomyelitis (EAE) by imparting severe inhibitory effects (red lines in the picture) on performance of T cells, B cells and macrophages, through secretion of soluble factors, such as IFN-γ, TNF-α, IL-10, HGF, IL-6, PGE2 and HO1. MSCs can also promote an anti-inflammatory effect through promotion of Treg proliferation (green line). In addition, MSCs also positively modulate the functions of astrocytes, oligodendrocytes, and neuronal axons. HGF: hepatocyte growth factor; PGE2: prostaglandin E2; HO1: heme oxygenase 1.

Clonal expansion of CD4^+^ Th1 and Th17 lymphocytes are key pathogenic features of relapses in RRMS, and few of the current drugs prevent relapses by immunosuppression. Several studies have acknowledged the universal immunosuppressive activities of MSCs, but the specific mechanisms in the context of autoimmune disorders still needs to be fully elucidated (Figure 1). Direct interactions (via cell–cell contact) of MSCs with T-cells *in vivo* leads to the arrest of Th1-cell proliferation, inhibition of CD8^+^-mediated cytotoxicity and generation of anti-inflammatory CD4^+^ regulatory T-cells [38]. As a consequence, impaired CD4^+^ T-cell activation translates into defective T-cell help for B-cell proliferation and differentiation to antibody-secreting cells. MSCs also inhibit pathogenic Th17 cells differentiation via IFN-γ-mediated suppressor of cytokine signaling 3 (SOCS3) activation [39].

Several soluble immunosuppressive factors, either produced constitutively by MSCs or released following cross-talk with target cells, have been reported, including NO and IDO [37]. IDO induces the depletion of tryptophan from the local environment, which is an essential amino acid for lymphocyte proliferation. MSC-derived IDO was reported as a requirement to inhibit the proliferation of IFN-γ producing Th1 cells and, together with prostaglandin E2 (PGE2), to block natural killer (NK)-cell activity [34]. In addition, IFN-γ, alone or in combination with TNF-α and IL-10, stimulates the production of chemokines by mouse MSCs that attract T-cells and stimulate the production of inducible nitric-oxide synthase (iNOS), which in turn inhibits T-cell activation through the production of NO [31]. It is worth noting that MSCs from IFN-γ receptor (IFN-γ-R1) deficient mice do not have immunosuppressive activity, which highlights the vital role of IFN-γ in the immunosuppressive function of MSC [31]. We conclude that MSCs have the potential to directly or indirectly inhibit disease-associated Th1, Th2, and Th17 cells as well as cytotoxic CD8^+^ lymphocytes but that many key questions regarding the, specificity, mechanistic basis, and predictable therapeutic value of these modulatory effects remain unanswered in the context of MS therapy.

**Keypoints:** Both mouse and human BM-MSCs cause Th17 cell anergy, inhibit CD8^+^ T cell mediated cytotoxicity, and promote Treg cell generation in EAE.

## 10. Immunomodulatory Action of MSCs in MS

Th17 and Tregs have been implicated as key players in MS, and a functional imbalance of these cell types is increasingly recognized as a key etiological factor in this disease progression. Tregs are a subpopulation of CD4, CD25, FoxP3 expressing T cells that are vital to keep the immune system in check, help avoid immune-mediated pathology, and contain unrestricted expansion of effector T-cell populations, which results in maintaining tolerance to self-antigens and disease remission. MSCs have been reported to induce the production of IL-10 by peripheral DCs, which, in turn, trigger the generation of Tregs [40,41]. In addition, *in vitro*, MSCs can directly induce the proliferation of Tregs through release of the immunomodulatory HLA-G isoform HLA-G5 [42]. Taken together, MSCs have the capacity to modulate the intensity of an immune attack in MS by inhibiting antigen-specific T-cell proliferation and cytotoxicity and promoting the generation of Tregs.

**Keypoints:** MSCs can exert anti-inflammatory effects by favoring the generation and proliferation of Th2 cells. MSc can also promote self-tolerance by inhibiting the dendritic cell ability to become antigen presenting cells.

Recent studies also suggest that activated T cells are not the only targets of MSCs. The striking disease attenuation seen after systemic administration of MSCs in EAE mice is thought to be in part mediated by the induction of peripheral tolerance [30] through the inhibition of dendritic cell maturation and function [43,44]. Since dendritic cells are the main antigen presenting cells for T-cell responses, MSC-mediated suppression of dendritic cell (DC) maturation would prohibit efficient antigen presentation and thus, the clonal expansion of autoreactive T-cells.

Soluble factors, such as transforming growth factor-beta1 (TGF-β1), hepatocyte growth factor (HGF), IL-10, PGE2, heme-oxygenase-1 (HO1), interleukin 6 (IL-6), and soluble HLA-G5, are constitutively produced by MSCs [29,42,43,45] or secreted in response to cytokines released by the target cells upon interacting with MSC, and mediate the immune modulating effects of MSCs.

The inflammatory cytokine IL6 appears to play a critical role in both the development of Th17 response and the inhibition of Treg functions. IL-6 is constitutively produced by MSCs that perform their effects in an autocrine or paracrine manner on the BM-MSCs themselves and on surrounding cells. In RRMS patients, functionally active Treg cells are unable to control the proliferation of autoreactive T effector cell (Teff) efficiently. This unresponsiveness is thought to be caused by accelerated production of IL-6, and elevated IL-6 receptor expression in CD4^+^ and CD8^+^ Teff cells. Exogenous IL-6 can change the kinetics of IL-6 production and induce a positive feedback loop in Teff from therapy-naïve MS patients [46]. Moreover, IL-6 signaling acts as a switch for MSC immune-modulation of macrophages. In the presence of IL-6, MSCs polarized macrophages towards M2 (alternatively activated macrophage phenotype) anti-inflammatory phenotype [47], while in the absence of IL-6, MSCs favored M1 (classically activated macrophage phenotype) polarization, which is characterized by IFN-γ, TNF-α, and CD40L expression.

Recent reports showed that MSCs derived from human embryonic stem cells alleviate clinical symptoms in a mouse EAE model, significantly superior to human bone-marrow-derived MSCs (BM-MSCs). Increased IL-6 production could enhance inflammation in EAE by directing mouse Th17 differentiation [48]. Human BM-MSCs plus the anti-human interleukin-6 antibody remarkably attenuated clinical symptoms in EAE mice compared to IL-6 antibody alone or BM-MSCs plus isotype control antibody [49]. Downregulation of IL-6 secretion in MSCs may provide a novel strategy to improve MSC-based therapy, and gene silencing technology as a potent weapon could be employed for this purpose. These data point to the complex nature of immune modulation by MSCs, and their implication in MS therapy needs to be analyzed carefully.

**Keypoints:** IL-6 is constitutively produced by MSCs. Higher levels of IL-6 secretion by BM-MSCs relative to human embryonic stem cell (hESC)-MSCs have been shown to contribute to the compromised ability of BM-MSCs to modulate EAE disease severity. Human BM-MSCs plus the anti-hIL-6 antibody remarkably attenuated clinical symptoms in EAE mice. These data point to the complex nature of immune modulation by MSCs in the inflammatory milieu.

HGF is thought to mediate the tolerogenic effects of MSC-stimulated in EAE [50], as systemic administration of HGF can ameliorate EAE by inducing tolerogenic dendritic cells (DCs). Administration of human MSC conditioned medium (hMSC-CM) containing HGF reduced CNS inflammation and clinical deficits in EAE. Interestingly MSCs derived from MS patients, exhibited downregulation of HGF genes expression and decreased secretion of IL-10 and TGF-β in supernatants, compared to MSC from healthy donors. These might explain the relatively dampened immunomodulatory and immunosuppressive activities of bone-marrow MSCs derived from MS patients [51]. Exogenous MSC can be devoid of this defect. Overall, HGF derived from MSC is a promising candidate for the treatment of MS and other T cell-mediated autoimmunity and neurodegeneration.

## 11. Interaction of Endogenous MSCs and the Inflammatory Environment in EAE Determines the Outcome of Autoimmune Response

Recent studies have demonstrated that MSCs themselves underwent autophagy in response to the inflammatory environment during their application for EAE treatment. Inflammatory cytokines, such as IFN-γ and TNF-α, induced autophagy and regulated the immunomodulatory function of MSCs [52]. Inhibition of autophagy in MSCs increased their immunosuppressive effects on T cell-mediated EAE [53]. These facts indicated that the interaction between MSCs and the inflammatory microenvironment determines the outcome of MSC-mediated therapy.

## 12. MSC-Derived Microvesicles (MVs) as Therapeutic Vehicle

MSCs can also function as attractive vehicles for cell and gene therapeutic applications. In particular, MSC-derived microvesicles (MVs), tiny vesicle like structures of cytoplasm surrounded by a membrane, can serve as a vehicle to transfer protein, coding and noncoding RNAs to target cells, altering the gene expression, proliferation, and differentiation of the recipient cells [54]. Several reports indicate that the regenerative effect of MSCs can be reproduced by MVs isolated from their culture medium [55]. In a rat model of unilateral acute kidney injury (AKI), the administration of MSCs derived-MVs promoted renal function recovery. MVs deliver RNA into injured tubular cells and induce HGF synthesis via RNA transfer, thereby facilitating tubular cell dedifferentiation and regeneration [56]. Other recent evidence implicated that the immunomodulatory effects of MSCs are also partially mediated by soluble MVs. MVs-based regeneration therapy represent a promising opportunity to develop novel cell-free therapy approaches that might evade the obstacles and risks associated with the use of native or engineered stem cells [57].

Since MSCs could impact the fate of surrounding cells and microenvironments via cell-cell contact and soluble factors, the question whether the inflammatory microenvironment and cytokines secreted by inflammatory cells or other cells localized within the CNS exert influence on the biological properties of MSCs and therapeutic effects of transplanted cells is of great importance for further study.

## 13. Clinical Trials in MS Using MSCs

Numerous clinical trials were conducted in the past several years, and encouraging results supported the feasibility and safety of MSC therapy [58]. In a phase II study, nine RRMS patients received intravenous infusion of BM-MSCs for six months, and the results indicated that patients treated with MSCs had a trend to lower cumulative number of gadolinium-enhancing lesions (GEL) on magnetic resonance imaging (MRI), while non-significant decrease of the frequency of Th1 cells in peripheral blood of MSCs treated patients was observed [59].

In another open-label phase 2a trial designed to assess the safety and efficacy of autologous MSCs as a feasible neuroprotective treatment for SPMS, ten patients with SPMS involving the visual pathways were recruited and received IV injection of autologous BM-MSCs, and six months after treatment, improvement in visual acuity and visual evoked response latency were observed [60]. These results are consistent with the neuroprotective effect of MSCs by promotion of endogenous oligodendrogenesis and remyelination [22]

In 2015, a Phase I neural stem cell clinical has been initiated with 20 RRMS patients, to study the safety and efficacy of autologous MSC derived neural progenitor cells. Intrathecal injections of expanded MSC-neural precursor cell (NPC) will be done over a six-month period, and patients will be followed for 27 months. The secondary objective of this trial is to measure the efficacy over a period of 36 months.

## 14. Induced Pluripotent Stem Cell (iPSCs)

In 2006, Takahashi and Yamanaka’s ground breaking research led to the generation of induced pluripotent stem cells (iPSCs) by reprogramming of mouse fibroblasts back into an embryonic stem cell-like state [13]. iPSCs were obtained by the over-expression of a combination of four genes: *Oct3/4*, *Sox2*, *c-Myc*, and *Klf4* in mouse fibroblasts. Subsequently, scientists succeeded in generating iPSCs from adult human dermal fibroblasts with the same four factors. iPSCs are very similar to ESCs with respect to their morphology, gene expression, self-renewal, telomerase activity, proliferation, and their capacity to differentiate into cell types of the all three germ layers both *in vitro* and in teratomas [61].

**Figure 2:**
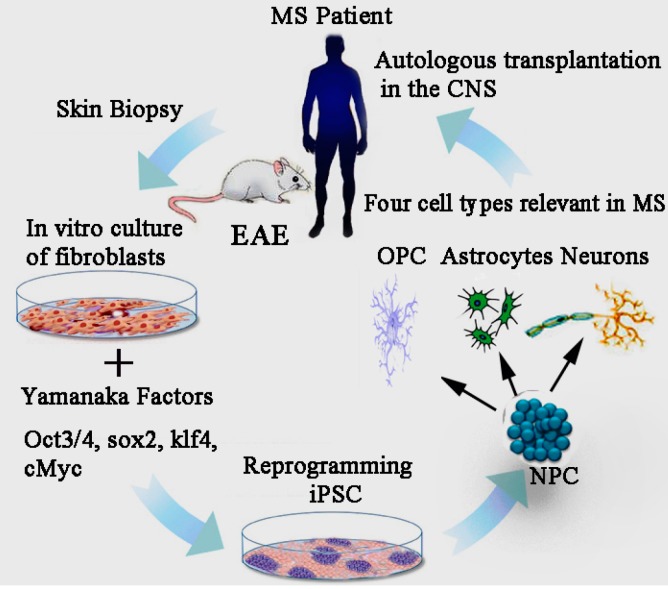
The scientific rationale behind human iPSC-based therapeutics. The underlying concept is that a patient’s own skin fibroblast cells could be epigenetically reprogrammed back into induced pluripotent stem cells. The primary dermal fibroblasts (**left** image) from biopsy samples can be reprogrammed into iPSCs (**bottom** image), which can be differentiated into NPCs, and subsequently into OPCs, neurons and astrocytes (**right** image), and then autologously transplanted back into the original individual, at a low risk of immune rejection. The future goal of the iPSC-based cell therapy is to use iPSCs-derivatives to develop personalized neuroregenerative and immunomodulatory therapies. MS: multiple sclerosis; NPC: neural precursor cells; OPC: oligodendrocyte precursor cells; iPSCs: induced pluripotent stem cells.

The iPSC technology is currently being used successfully to generate different neuronal cells types which are capable of producing fully functional cells. Specific patient iPSC-derived cells can recapitulate many pathological features of disease at the molecular level, and thus offers a unique platform to model and study many aspects of neurodegenerative “disease-in-a-dish” (Figure 2). This could ultimately lead to new insights in disease pathology and developments of novel drugs. For example, patient-specific iPSCs are proving useful and efficient in distinguishing individuals that are likely to respond to new therapeutics, and seem to be accurately predicting toxicity and efficacy in comparison to animal models [62] The recent improvements in generating autologous iPSCs from almost any somatic cell type has brought autologous cell therapies at the fore front of clinical research. In 2011, researchers differentiated mouse iPSCs successfully into neural precursor cells (NPCs) and oligodendrocyte precursor cells (OPCs) that could develop into mature, myelinating oligodendrocytes *in vitro* as well as *in vivo* [42]. This paves the way for autologous cell replacement, since iPSCs are obtained from a patient’s own tissues, there will be minimal risk of immunological rejection [63]. As an emerging therapeutic method for MS, iPSCs need to be cultured *in vitro* to harvest fully differentiated, expandable and functional NPCs or OPCs for implantation purposes. [64]

## 15. Effect of iPSCs Derived Neural Precursor Cells (NPCs) in EAE

In order to assess the therapeutic potentials of iPSCs in MS, mouse iPSC-derived neural precursor cells (miPSC-NPCs) have been tested for its ability to generate OPC and remyelinate in the mouse EAE model. The miPSC-NPCs transplanted intrathecally in presymptomatic mice with EAE significantly ameliorated clinical and pathological features of EAE [65]. However, the immediate efficacy was found to be mostly due to the neuroprotective effect of the transplanted miPSC-NPCs, and not through remyelination. The neuroprotective effects were in part through the secretion of leukaemia inhibitory factor (LIF) that promoted viability of endogenous OPC and remyelination by endogenous oligodendrocytes [65].

## 16. Mechanisms of Action of iPSC–Neural Stem Cells (NSCs): Candidate Factors

Recent studies have shown that transplanted miPSC-NPCs exert the neuroprotective effect not through cell replacement, but through the secretion of leukaemia inhibitory factor (LIF) that promotes survival, differentiation and the remyelination capacity of both endogenous oligodendrocyte precursors and mature oligodendrocytes in the EAE model [65]. LIF, is a member of the IL-6 cytokine family, and is implicated significantly in the pathophysiology of MS. Recent evidence demonstrates a crucial role of LIF in neuroprotection and axonal regeneration as well as the prevention of demyelination [66,67]. Levy *et al.* 2015, demonstrated that expression of the LIF receptors was strongly increased on immune cells of RRMS patients, whereas, the secreted LIF levels from immune cells of RRMS patients were downregulated compared to healthy controls. This immune dysregulation in patients with RRMS is thought to be associated with insufficient remyelination and neurogenesis in MS lesions [68]. LIF treatment potently boosted the number of regulatory T cells (Tregs) in MS patients with low serum levels of IL-6 [69]. Also, a balance between LIF and IL-6 levels is thought to be crucial for maintaining the balance between pathogenic Th17 cells vs beneficial Treg cells. In the EAE model a shift in the LIF/IL-6 balance in favor of LIF by overexpression of LIF, increased the number of Tregs in the CNS during active autoimmune responses and reduced the clinical signs of EAE. Overall, these data show that increasing the local levels of LIF in the CNS can down-regulate the autoimmune response. The process of neuroinflammation attracts miPSC-NPCs to the site of myelin damage and fosters such cells to secrete LIF upon inflammatory stimuli. This in turn has been shown to promote endogenous OPC survival and maintain functionality of mature oligodendrocytes. These studies provide further impetus for the use of iPSC-NPCs as a novel treatment for MS and other autoimmune diseases [69]. Exogenously infused LIF has limited ability to cross the blood–brain barrier and has pleiotropic actions outside the CNS making the NPC an ideal choice for delivering LIF. The miPSC-NPCs also appear to help preserve the BBB permeability and integrity and restrict the continuous CNS recruitment of encephalitogenic blood-borne inflammatory cells [70].

## 17. iPSCs Derived Oligodendrocyte Precursor Cells (OPCs) as a Therapeutic Candidate for MS

An effective therapeutic strategy for the treatment of demyelinating diseases such as multiple sclerosis would require the replacement of glial precursor cells, namely OPCs. Subsequently, a number of strategies have been developed for the cell-based repair of demyelinated lesions in the CNS [71,72]. In particular, human OPCs capable of maturation and myelination have been derived from both fetal and adult human brain tissue [73,74,75], as well as from human embryonic stem cells (hESCs) [76,77,78], and have proven effective in experimental models of both congenitally dysmyelinated [74,79,80] and adult demyelinated brain [81]. Yet the successes in efficacy, has been complicated by massive immune rejection seen with allogenic cells, and thus far hindered the use of allogeneic human cells as transplant vectors. The issue of transplant rejection is especially problematic in case of MS, in which the inflammatory processes underlying these disorders can present an intrinsically hostile environment to any allogeneic grafts [82].

To establish a potential autologous source of these cells, Wang *et al.* successfully generated OPCs from three human induced pluripotent stem cell (hiPSC) lines [83]. These highly enriched oligodendrocyte lineage transcription factor 2/platelet derived growth factor receptor alpha/NK2 homeobox 2/sex determining region Y-box 10 (OLIG2^+^/PDGFRα^+^/NKX2.2^+^/SOX10^+^) human OPCs, efficiently differentiated into both myelinogenic oligodendrocytes and astrocytes, *in vitro* and *in vivo*, and were able to rescue a hypomyelinated mouse model. Neonatally engrafted human iPSC-OPCs robustly myelinated the brains of myelin basic protein (MBP)-deficient *shiverer* mice, and significantly increased the life span of these mice. The efficiency of myelination by human iPSC-OPCs was higher than that previously observed using fetal tissue-derived OPCs, and no tumors from these grafts were noted as long as 36 weeks after transplant. This seminal study has provided great impetus to the feasibility of using human iPSC-derived OPCs in treating MS. The success of their protocol in all three lines used in this study, suggest its broad range applicability, while the high efficiency gliogenesis afforded by this strategy indicates its robust nature. Further studies are urgently needed regarding the development and functional characterization of OPCs derived from different types of MS patients. It remains to be seen if these OPCs retain the same functionality as iPSC-OPCs derived from healthy subjects. In 2014, Douvaras *et al.* developed a rapid, highly efficient and robust protocol by which they generated myelinating oligodendrocytes from primary progressive MS, through iPSC technology [46]. The protocol involved generation of iPSCs from skin biopsies, followed by neural induction by dual Sme mothers against decapentaplegic (SMAD) inhibition and exposure to retiniods from the beginning of differentiation, which led to generation of Olig2-expressing progenitors and a high number of OPCs in about 11 weeks. So far we have not come across any reports on the effects of iPSC-derived OPCs in the EAE model.

Although MS results in death of mature oligodendrocytes (OLs), there is ample evidence that endogenous OPCs proliferate robustly in an attempt to differentiate and remyelinate the brain during relapses in early disease. However, these OPC are inhibited from terminal differentiation, and therefore remyelination fails. Later in the disease course, the OPC pool gradually disappears, and thus cell replacement could be of great benefit at that stage. The success of cell therapy, however, relies on the fact that exogenously transplanted OPCs would be able to terminally differentiate and mature to make myelin. Further research is needed in this area to characterize the fate of transplanted OPCs in the inflammatory environment.

## 18. Modelling MS Pathology though the iPSC Platform

Human dermal fibroblasts from RRMS patients have been reprogrammed to pluripotent state via retroviral transduction. The MS-iPSCs can successfully differentiate into mature astrocytes, oligodendrocytes and neurons with normal karyotypes [84]. Although MS-iPSC-derived neurons presented some differences in their electrophysiological characteristics as compared to the control hESCs, they did exhibit properties of functional neurons, with robust resting membrane potentials, large fast tetrodotoxin-sensitive action potentials and voltage-gated sodium currents. These results demonstrated for the first time that iPSCs derived from disease-state patients can be generated and differentiated into mature neural lineages. In 2014, Douvaras *et al.* reported the generation of iPSCs from skin biopsies from four PPMS patients and their efficient differentiation to functional oligodendrocytes, as demonstrated by *in vivo* myelination in the *shiverer* mouse. PPMS-OPCs also differentiated into mature oligodendrocyte which formed dense compact myelin resembling normal myelin in EAE mice, as efficiently as iPSC-derived OPCs from healthy subjects [45]. The general protocol for induction of iPSCs into OPCs and mature oligodendrocyte *in vitro* is very inefficient and required 120–150 days in culture. An improved protocol uses at least 75 days to harvest the OPCs [45]. Given the rapid progress in this emerging field, iPSCs may demonstrate great potential in clinical use for MS treatment.

## 19. Risks and Disadvantages with the Use of MSCs and iPSCs in Clinical Applications

The epigenetic memory of iPSCs is a signature acquired from parental cells and the reprogramming process, which restricts the ability of iPSCs to differentiate and form cells from alternative lineages and creates predisposition to redifferentiate into their original cell type [85]. These iPSC epigenetic signatures can be transmitted to their progeny even after differentiation and may affect the function of iPSC-derived cells [86]. Due to selective pressures of culture conditions and the consequences of reprogramming to produce iPSCs, including genetic mutations and overexpression, it will be important to improve reprogramming protocols to overcome these hurdles and generate genetically stable iPSCs.

## 20. Conclusions

Stem cell therapy using MSCs or iPSCs shows great potential as treatment for MS. However, there are many issues and limitations that need to be resolved. The specific cell stage to be transplanted, proper characterization of the cell type to be administered, *in vivo* fate of transplanted cells in different inflammatory models, dose, route of administration, duration of therapeutic effect and genomic stability of stem cells need further exploration and quantification. Patient-derived iPSCs also represent a novel tool in modeling MS pathology enabling the confirmation of positive responders to new pharmacotherapy and implementation of patient-specific therapeutic managements.
